# Loss of GIPR in LEPR cells impairs glucose control by GIP and GIP:GLP-1 co-agonism without affecting body weight and food intake in mice

**DOI:** 10.1016/j.molmet.2024.101915

**Published:** 2024-03-14

**Authors:** Seun Akindehin, Arkadiusz Liskiewicz, Daniela Liskiewicz, Miriam Bernecker, Cristina Garcia-Caceres, Daniel J. Drucker, Brian Finan, Gerald Grandl, Robert Gutgesell, Susanna M. Hofmann, Ahmed Khalil, Xue Liu, Perla Cota, Mostafa Bakhti, Oliver Czarnecki, Aimée Bastidas-Ponce, Heiko Lickert, Lingru Kang, Gandhari Maity, Aaron Novikoff, Sebastian Parlee, Ekta Pathak, Sonja C. Schriever, Michael Sterr, Siegfried Ussar, Qian Zhang, Richard DiMarchi, Matthias H. Tschöp, Paul T. Pfluger, Jonathan D. Douros, Timo D. Müller

**Affiliations:** 1 Institute for Diabetes and Obesity, Helmholtz Munich, Neuherberg, Germany; 2 German Center for Diabetes Research (DZD), Neuherberg, Germany; 3 Helmholtz Diabetes School, Helmholtz Diabetes Center, Munich, Germany; 4 Department of Physiology, Faculty of Medical Sciences in Katowice, Medical University of Silesia, Poland; 5 Institute of Physiotherapy and Health Sciences, Academy of Physical Education, Katowice, Poland; 6 Neurobiology of Diabetes Research Unit, Germany; 7 Medizinische Klinik und Poliklinik IV, Klinikum der Universität, Ludwig-Maximilians-Universität München, Munich, Germany; 8 Lunenfeld-Tanenbaum Research Institute, Mount Sinai Hospital, University of Toronto, Toronto, ON, Canada; 9 Novo Nordisk Research Center Indianapolis, Indianapolis, IN, USA; 10 Institute of Diabetes and Regeneration Research, Helmholtz Munich, Neuherberg, Germany; 11 Medical Clinic and Polyclinic IV, Ludwig-Maximilians University of München, Munich, Germany; 12 RU Adipocytes & Metabolism, Helmholtz Diabetes Center, Helmholtz Zentrum München, German Research Center for Environmental Health GmbH, 85764 Neuherberg, Germany; 13 Department of Chemistry, Indiana University, Bloomington, IN, USA; 14 Division of Metabolic Diseases, Department of Medicine, Technical University Munich, Munich, Germany; 15 Helmholtz Munich, Neuherberg, Germany; 16 Division of Neurobiology of Diabetes, TUM School of Medicine, Technical University of Munich, Munich, Germany; 17 Walther-Straub-Institute for Pharmacology and Toxicology, Ludgwig-Maximilians-University Munich, Germany

**Keywords:** Obesity, Type 2 diabetes, GIP, GLP-1, GIPR:GLP-1R co-agonism

## Abstract

**Objective:**

The glucose-dependent insulinotropic polypeptide (GIP) decreases body weight via central GIP receptor (GIPR) signaling, but the underlying mechanisms remain largely unknown. Here, we assessed whether GIP regulates body weight and glucose control via GIPR signaling in cells that express the leptin receptor (*Lepr*).

**Methods:**

Hypothalamic, hindbrain, and pancreatic co-expression of *Gipr* and *Lepr* was assessed using single cell RNAseq analysis. Mice with deletion of *Gipr* in *Lepr* cells were generated and metabolically characterized for alterations in diet-induced obesity (DIO), glucose control and leptin sensitivity. Long-acting single- and dual-agonists at GIPR and GLP-1R were further used to assess drug effects on energy and glucose metabolism in DIO wildtype (WT) and *Lepr-Gipr* knock-out (KO) mice.

**Results:**

*Gipr* and *Lepr* show strong co-expression in the pancreas, but not in the hypothalamus and hindbrain. *DIO Lepr-Gipr* KO mice are indistinguishable from WT controls related to body weight, food intake and diet-induced leptin resistance. Acyl-GIP and the GIPR:GLP-1R co-agonist MAR709 remain fully efficacious to decrease body weight and food intake in DIO *Lepr-Gipr* KO mice. Consistent with the demonstration that *Gipr* and *Lepr* highly co-localize in the endocrine pancreas, including the β-cells, we find the superior glycemic effect of GIPR:GLP-1R co-agonism over single GLP-1R agonism to vanish in *Lepr-Gipr* KO mice.

**Conclusions:**

GIPR signaling in cells/neurons that express the leptin receptor is not implicated in the control of body weight or food intake, but is of crucial importance for the superior glycemic effects of GIPR:GLP-1R co-agonism relative to single GLP-1R agonism.

## Highlights

• Diet-induced obese (DIO) mice with deletion of *Gipr* in *Lepr* neurons show normal body weight and food intake relative to WT controls.
• Acyl-GIP increases cFos neuronal activation in a subset of hypothalamic Pomc neurons, confirming that GIP acts centrally to decease feeding.
• Acyl-GIP and the GIPR:GLP-1R co-agonist MAR709 remain fully efficacious to decrease body weight and food intake in DIO *Lepr-Gipr* KO mice.
• The superior glycemic effect of GIPR:GLP-1R co-agonism over single GLP-1R agonism vanishes in *Lepr-Gipr* KO mice.
• GIPR in *Lepr* neurons is not involved in global GIP anti-obesogenic effect, but GIPR in pancreatic *Lepr* cells mediates glycemic benefits.

## 1 Introduction

Co-agonists at the receptors for glucagon-like peptide-1 (GLP-1) and the glucose-dependent insulinotropic polypeptide (GIP) are among the best-in-class drugs to treat obesity and type 2 diabetes [1]. GIPR:GLP-1R co-agonists improve body weight and glucose control with superior efficacy relative to single GLP-1R agonists in preclinical [1,2] and clinical studies [3,4]. However, the mechanisms of how GIP regulates systemic metabolism remain largely unknown [[5], [6], [7]]. A long-acting fatty acid-acylated GIPR agonist (acyl-GIP) was recently shown to decrease body weight and food intake in diet-induced obese (DIO) mice, and these effects vanish in mice with either Nestin-Cre-mediated neuronal loss of *Gipr* [8], or with deletion of *Gipr* specifically in inhibitory (*Vgat*-expressing) GABAergic neurons [9]. Hypothalamic and hindbrain activation of GIPR emerged as a potential mode of action for the weight lowering efficacy of GIP, as evidenced by the demonstration that GIPR agonists increase cFos neuronal activity in these areas [[8], [9], [10], [11], [12]], and that chronic intracerebroventricular (icv) infusion of acyl-GIP into the lateral ventricle decreases body weight and food intake in wildtype mice, but not in mice with neuronal loss of *Gipr* [8]. The role of hypothalamic and hindbrain GIPR in regulating energy metabolism is further corroborated by studies showing that DREADD-mediated activation of hypothalamic and hindbrain GIPR neurons decreases food intake in mice [10,11], and that the superior body weight lowering effect of the GIPR:GLP-1R co-agonist MAR709 over a pharmacokinetically-matched GLP-1 control vanishes in mice with deletion of *Gipr* in either the entire CNS [8], or specifically in GABAergic neurons [9]. Delineating the spatial contribution of GIPR cells for the anti-obesity effects of acyl-GIP is key for a better understanding of this important new class of drugs. Of particular importance might be cells that co-express *Gipr* and the leptin receptor (*Lepr*). Central infusion of GIP was recently shown to decrease hypothalamic leptin sensitivity in mice, while central immunoneutralization of GIPR protects from HFD-induced leptin resistance [13]. Leptin signaling is of central importance for body weight control [14], and the leptin receptor is expressed in multiple brain areas and peripheral tissues known to be targeted by GIP, including the hypothalamus and the hindbrain. Accordingly, we here aimed to assess whether GIP and GIPR:GLP-1R co-agonism require GIPR signaling in cells/neurons that express the leptin receptor (*Lepr*) to regulate body weight, food intake and glucose metabolism.

## 2 Results

### 2.1 Co-expression of Gipr and Lepr in the hypothalamus

**Figure 1:**
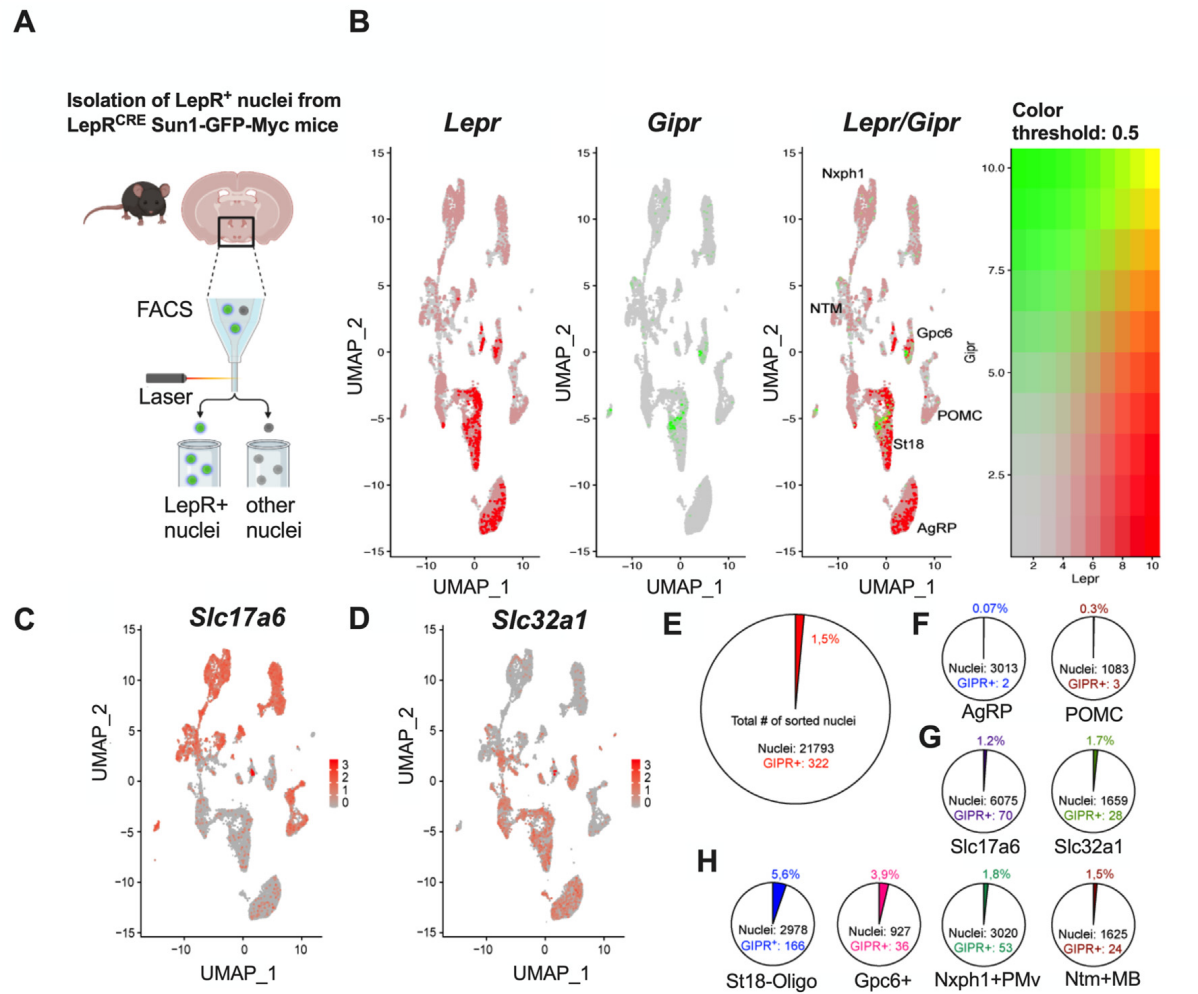
**Single nuclei (sn)RNA-seq analysis of *Lepr* positive cells in the murine hypothalamus.** Schematic for the selective isolation of hypothalamic Lepr positive nuclei from *Lepr*-Cre Sun1-GFP-Myc reporter mice using fluorescence-activated cell sorting (FACS) **(A)**. Feature plots of snRNAseq for cells expressing *Lepr*, *Gipr,* and *Lepr/Gipr***(B)**. The overlay panel reveals low abundance for *Gipr* that appears restricted to the clusters *St18*, *Gpc6*, *Nxph1* and *Ntm*. Gipr is largely absent from the *Pomc* and *Agrp* clusters. Feature plots for the glutamatergic **(C)** and GABAergic **(D)** neuronal markers Slc17a6 and Slc32a1, respectively. Pie charts of total and relative numbers of *Gipr* positive nuclei **(E**–**H)** in all clusters combined **(E)**, *Agrp* and *Pomc* clusters **(F)**, glutamatergic (Slc17a6) vs. GABAergic (Slc32a1) neurons **(G)** and clusters with low but detectable *Gipr* abundance, defined via their distinct marker transcripts Suppression of tumorigenicity 18 (*St18*), Glypican 6 (*Gpc6*), Neurexophilin 1 (*Nxph1*) and Neurotrimin (*Ntm*) **(H)**.

To assess whether *Gipr* is expressed in hypothalamic *Lepr* cells, we employed a L*epr-Cre* driven murine Rosa26^Sun1-GFP-Myc^ reporter model that allows for the selective isolation and characterization of *Lepr* nuclei by FACS and snRNA sequencing (Figure 1A). Consistent with previous data [15], we found only limited co-expression of *Gipr* and *Lepr* cells of the hypothalamus, with co-expression largely restricted to few, distinct *Lepr* clusters represented by key marker genes, namely *Suppression of tumorigenicity 18* (*St18*), *Glypican 6* (*Gpc6), Neurexophilin 1* (*Nxph1*) and *Neurotrimin* (*Ntm*) (Figure 1B). *Gipr* showed little co-expression with agouti-related peptide (*Agrp)* or proopiomelanocortin (*Pomc)*, or with markers indicative of glutamatergic (*SLC17a6*) and GABAergic (*SLC32a1*) neurons (Figure 1C,D). *Gipr* was expressed in a total of ∽1.5% of hypothalamic *Lepr* cells (Figure 1E). In the *Agrp* and *Pomc* clusters, only 0.07% and 0.3% of the cells expressed *Gipr*, respectively (Figure 1F). *Gipr* mRNA was detected in 1.2% of glutamatergic (*Slc17a6*) cells and in 1.7% of GABAergic (*Slc32a1)* cells (Figure 1G). Modest levels of co-expression were detected in the clusters *St18* and *Gpc6*, with 5.8% and 3.9%, followed by the clusters *Nxph1* with 1.8% and *Ntm* with 1.5% (Figure 1H).

**Figure S1:**
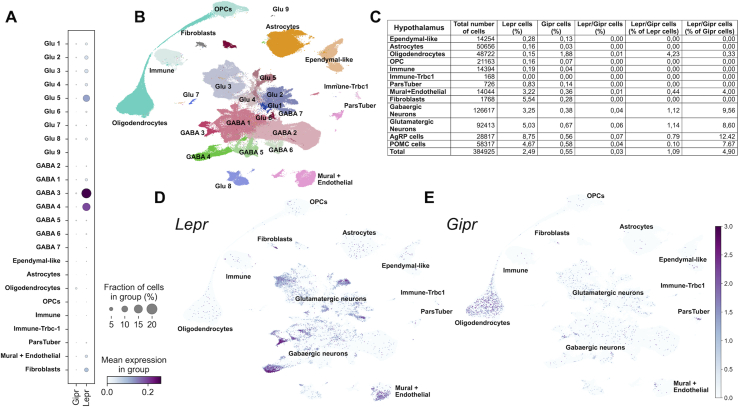
Co-expression of *Lepr* and *Gipr* based on the HypoMap Repository.

We also validated the abundance of *Gipr* expression in hypothalamic *Lepr* cells in the previously published HypoMap repository, which summarizes several published datasets on hypothalamic single cell RNA sequencing from mice [15]. In HypoMap, *Gipr* expression is found in ∽1% of the hypothalamic *Lepr* cells. Of those, ∽19% expressed *Agrp* (0.8% of *Lepr*^*+*^ *Agrp* cells), ∽25% *Pomc* (1% of *Lepr*+ *Pomc* cells), ∽18% *Slc17a6* (1.3% of *Lepr*+ *Slc17a6* cells) and ∽42% *Slc32a1* (0.9% of *Lepr*+ *SLC32a1* cells) (Supplementary Figure 1A-E). Collectively, these data indicate that *Gipr* and *Lepr* show only limited co-expression in the hypothalamus, with only a fraction (<1%) of AGRP and POMC neurons expressing both receptors.

### 2.2 Co-expression of Gipr and Lepr in the hindbrain

**Figure 2:**
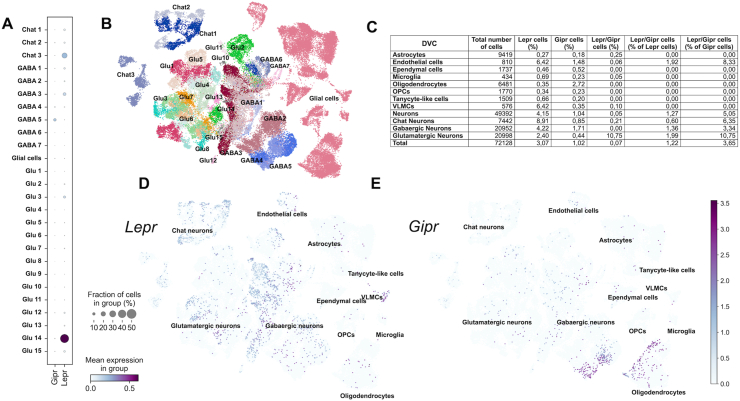
**Single cell (sc)RNAseq analysis of *Gipr* and *Lepr* in the hindbrain.** Expression of *Lepr* and *Gipr* in Chat, GABAergic and Glutamatergic neuron clusters in the DVC **(A)**, UMAP visualization of those clusters **(B)** and the percentage of cells expressing both receptors in DVC cell types **(C)**. UMAP visualization of the relative expression of *Lepr***(D)** and *Gipr***(E)** in DVC cells.

We similarly investigated the co-expression profile of Gipr and Lepr in a previously published scRNAseq repository for the dorsal vagal complex (DVC) [16]. In this dataset, Gipr and Lepr show abundant expression in the DVC, but clusters of neurons expressing high levels of Lepr do not express high levels of Gipr and vice-versa (Figure 2A,B), indicating that like the hypothalamus, there is very limited Gipr and Lepr co-expression in the DVC. Consistent with this, expression of Gipr is found in ∼1.2% of Lepr expressing cells (Figure 2C), and while Gipr expression is highly localized to GABAergic neurons, Lepr expression is more evenly distributed across all cell types in the DVC (Figure 2D–E).

### 2.3 Co-expression of Gipr and Lepr in the pancreas

**Figure 3:**
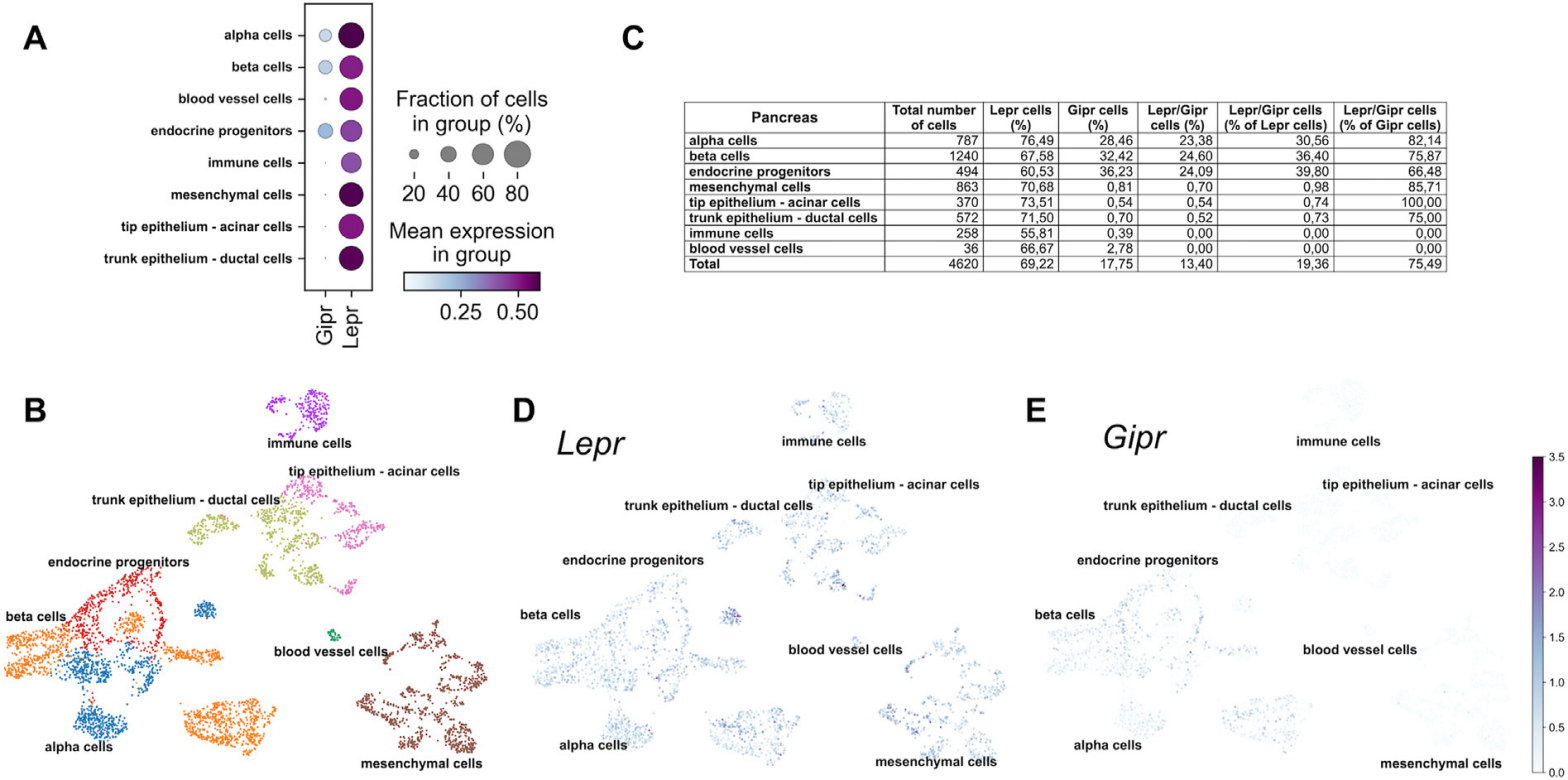
**Single cell (sc)RNAseq analysis of *Gipr* and *Lepr* in the embryonic murine pancreas.** Expression of *Lepr***(A)** and *Gipr***(B)** and percentage of cells expressing *Gipr, Lepr,* or both receptors within identified clusters **(C)** in the pancreas of mice at the embryonic ages E12.5 - E18.5 integrated into one data set [17].

The cellular distribution of pancreatic *Gipr* and *Lepr* expression was depicted from a published scRNA-seq repository, in which mouse pancreatic cells at the embryonic ages E12.5, E13.5, E14.5, E15.5 and E18.5 were integrated into a single dataset [17]. In contrast to *Lepr*, which is ubiquitously expressed in all cell types of the embryonic pancreas (∽69% of all cells), ∽95% of the *Gipr* positive cells correspond to the endocrine pancreas, with only scarce expression of *Gipr* in the exocrine pancreas (∽1%), blood vessels (∽2.8%), immune cells (∽0.4%) and mesenchymal cells (0.8%) (Figure 3A–E). In the embryonic pancreas, ∽75% of the *Gipr* expressing cells co-expressed the leptin receptor, with *Lepr* being expressed in ∽82% of the *Gipr* positive alpha cells and in ∽76% of the *Gipr* positive beta cells (Figure 3C).

### 2.4 GIPR agonism activates cFos expression in a subset of POMC neurons

**Figure 4:**
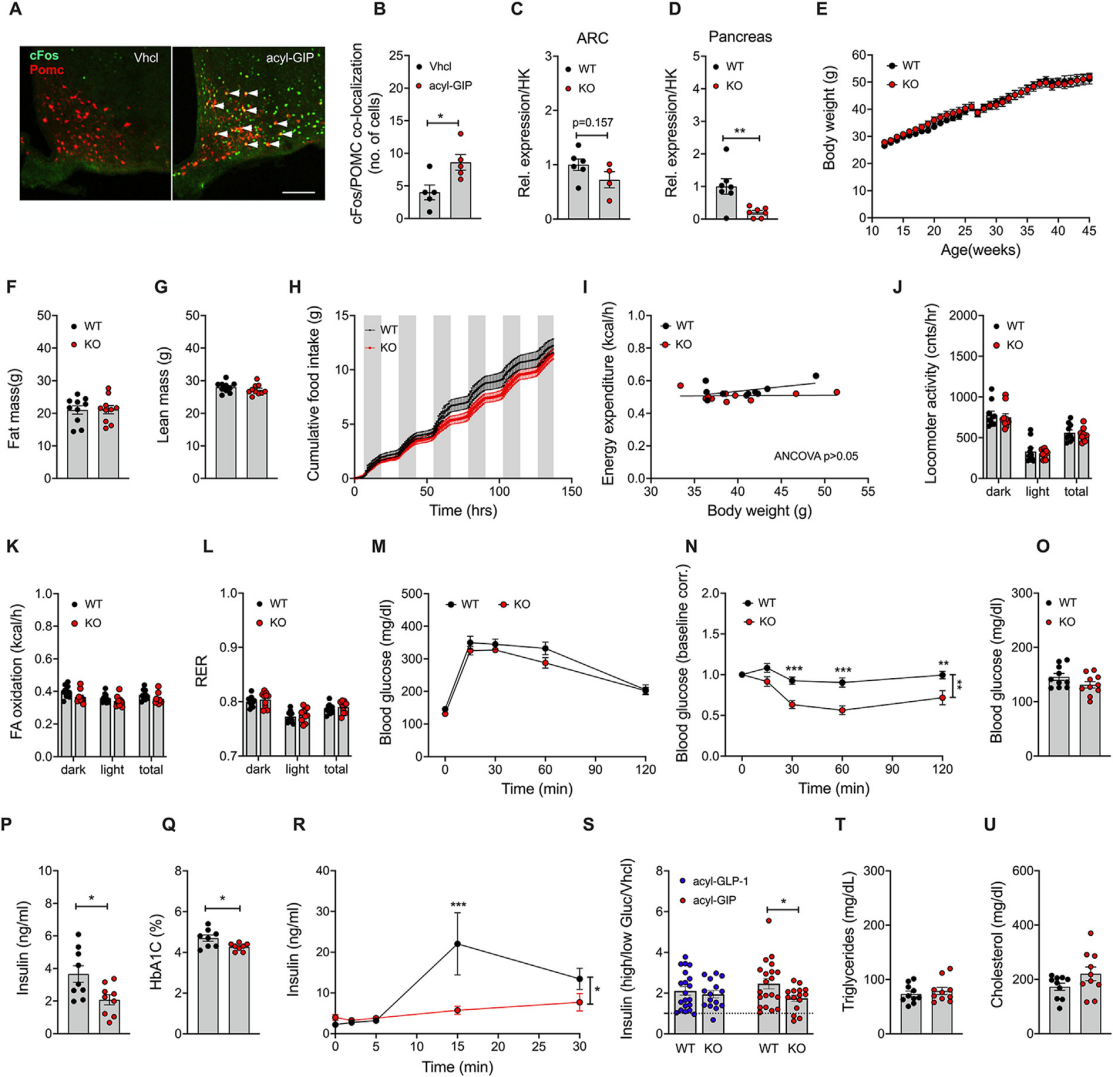
**Metabolic phenotype of HFD-fed male *Lepr*-*Gipr* KO mice.** Representative image **(A)** and quantification **(B)** of cFos in the hypothalamic arcuate nucleus (ARC) of 19-wk old HFD-fed POMC-GFP mice treated with a single s.c. bolus of acyl-GIP (30 nmol/kg) (n = 5 each group, scale bar 100 μm). Expression of *Gipr* in the ARC (n = 4–6 each group) **(C)** and pancreas of 24–32-wk old mice (n = 7 each group) **(D)**. Body weight **(E)** and body composition of 40-wks old mice (n = 10 each group) **(F, G).** Cumulative food intake **(H)**, energy expenditure **(I)**, locomoter activity **(J)**, fatty acid oxidation **(K)**, and respiratory exchange ratio **(L)** of 27-wk old mice (n = 10 each group). Intraperetoneal (i.p.) glucose tolerance of 40-wk old mice (n = 10 each group) **(M)** and i.p. insulin tolerance in 47-wk old mice (n = 10 each group) **(N)**. Fasting levels of blood glucose in 40-wk old mice (n = 10 each group) **(O)**, and fasting insulin in 47-wk old mice (n = 9 each group) **(P).** HbA1c in 42-wk old mice (n = 8 each group) **(Q)** in 40-wk old mice (n = 10 each group), and oral glucose-stimulated insulin secretion in 31-wks old mice (n = 8 each group) **(R)**. Glucose-stimulated insulin secretion in pancreatic islets isolated from 16 to 20-wks old mice and treated with acyl-GLP-1 (50 nM) or acyl-GIP (50 nM) for 45 min under conditions of high (20 mM) glucose (n = 15–20 mice each group). Plasma levels of triglycerides **(S)**, cholesterol **(T)**, and NEFA **(U)** in 40-wk old mice (n = 10 each group). Data represent means ± SEM. Asterisks indicate ∗ p < 0.05, ∗∗p < 0.01 and ∗∗∗p < 0.001. Longitudinal data **(E, H, M, N and R)** were analyzed using 2-way ANOVA with time and genotype as co-variables and Bonferroni post-hoc analysis for individual time-points. Bar graphs **(B-D, F, G, J-L, O-Q and S–U)** were analyzed using 2-tailed, 2-sided ttest. Data in **(J)** were analyzed using ANCOVA with body weight as co-variate as previously suggested [24,25]. Panel **A** is a representative example of N = 5 biological replicates. Data points in panels **B-D, F,G, I-L, O-Q and S–U** represent independent biological replicates.

**Figure S2:**
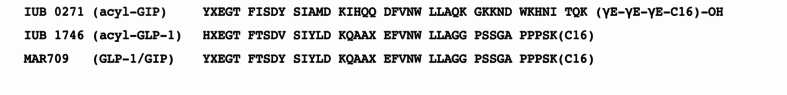
Amino acid sequence of the used peptides. Amino acid sequence and fatty acid acylation of acyl-GIP (IUB0271), acyl-GLP-1 (IUB1746) and the GIPR:GLP-1R co-agonist MAR709.

Based on our previous data showing that acyl-GIP increases cFos neuronal activation in the hypothalamus [8,9], and that *Gipr* is expressed in a small subset of POMC neurons [11,15], we assessed whether GIP activates cFos neuronal activity in POMC neurons by treating HFD-fed POMC-GFP mice with a single s.c. bolus of a validated fatty acylated GIP (Supplementary Figure 2) [8,18]. Consistent with our previous data [8,9], we found acyl-GIP to increase cFos neuronal activation in the arcuate nucleus (ARC), with significant co-localization in a subset of POMC neurons (Figure 4A,B). Based on this observation, and the well-established role of leptin in regulating food intake via stimulation of POMC neuronal activity [19], we next assessed whether GIPR agonism affects metabolism via GIPR signaling in cells/neurons that express the leptin receptor. Mice with specific deletion of *Gipr* in cells that express the leptin receptor were generated by crossing *Gipr*^*flx/flx*^ mice [20,21] with mice that express *Cre* recombinase under control of the *Lepr* promoter [22]. Consistent with the demonstrated co-expression pattern of *Gipr* and *Lepr* in the hypothalamus (Figure 1B–H) and the pancreas (Figure 3A–E), we found expression of *Gipr* only marginally decreased in the *Gipr* enriched ARC (p > 0.05), but decreased by 79% (p < 0.01) in the pancreas of *Lepr-Cre Gipr* KO mice (Figure 4C,D).

### 2.5 Mice with lack of Gipr in Lepr cells show impaired insulin secretion, but improved insulin sensitivity without alterations in body weight or food intake

**Figure 5:**
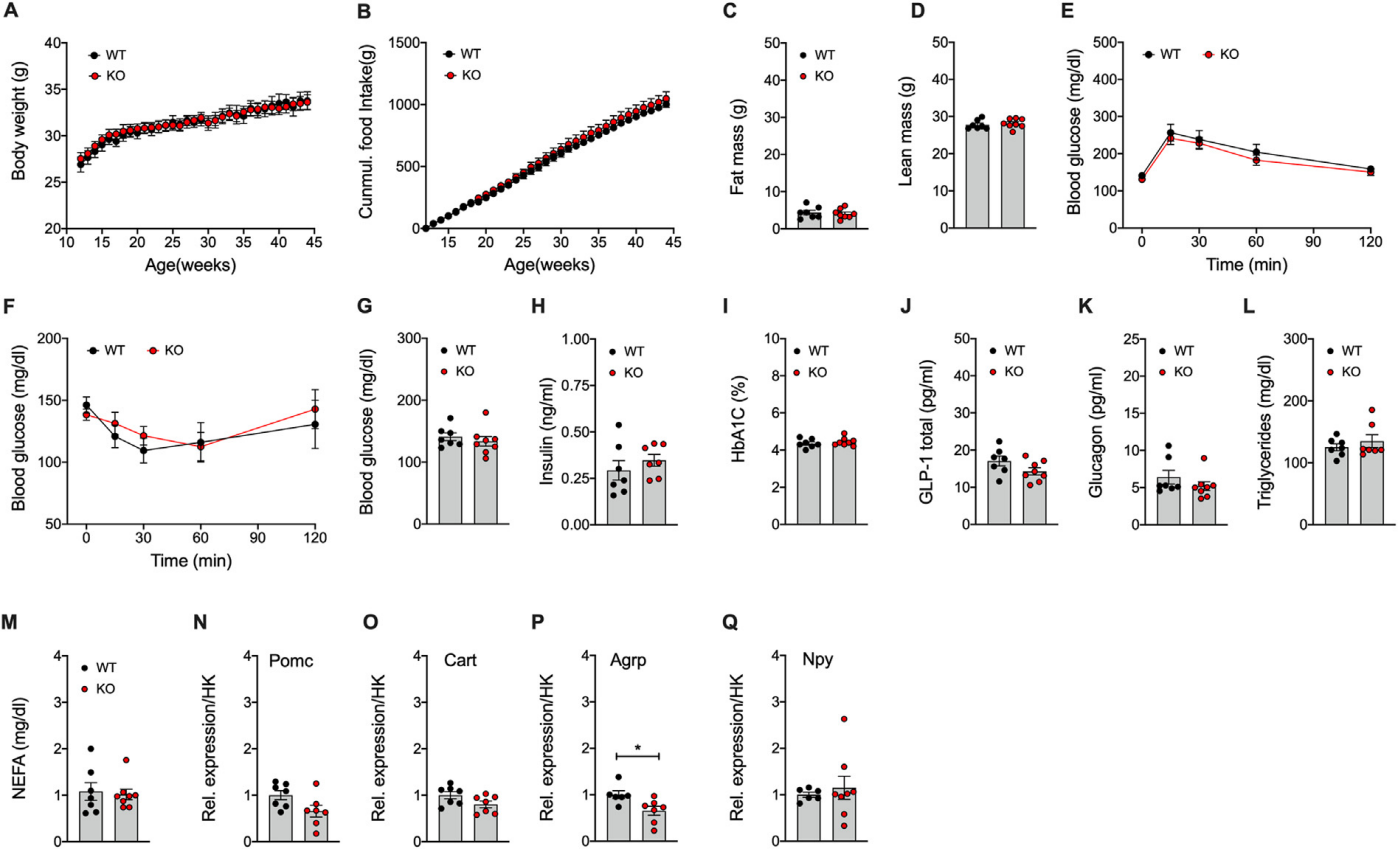
**Metabolic phenotype of chow-fed male *Lepr*-*Gipr* KO mice.** Body weight **(A)** and food intake **(B)** of male *Lepr* Cre *Gipr* KO mice and wildtype controls (n = 7–8 each group). Body composition **(C, D)** at the age of 45-wks (n = 7–8 each group). Intraperetoneal glucose tolerance at the age of 46-wks (n = 7–8 each group) **(E)** and i.p. insulin tolerance at the age of 49-wks (n = 7–8 each group) **(F)**. Fasting levels of blood glucose in 46-wk old mice (n = 7–8 each group) **(G)** and of insulin in 48-wk old mice (n = 7 each group) **(H)**. HbA1c **(I)** and fasting level of total GLP-1 **(J)** and glucagon **(K)** in 43-wk old mice (n = 7–8 each group). Plasma levels of triglycerides **(L)** and NEFA **(M)** in 47-wk old mice (n = 7–8 each group). Hypothalamic expression of *Pomc*, *Cart*, *Agrp* and *Npy* in 51-wk old mice (n = 6–8 each group) **(M**–**Q)**. Data represent means ± SEM. Asterisks indicate ∗ p < 0.05. Longitudinal data **(A, B, E, F)** were analyzed using 2-way ANOVA with time and genotype as co-variables and Bonferroni post-hoc analysis for individual time-points. Bar graphs **(C, D, G-Q)** were analyzed using 2-tailed, 2-sided ttest. Cumulative food intake **(B)** was assessed per cage in single or double-housed mice (n = 7–8 each group). Data points in panels **C,D,G-Q** represent independent biological replicates.

**Figure S3:**
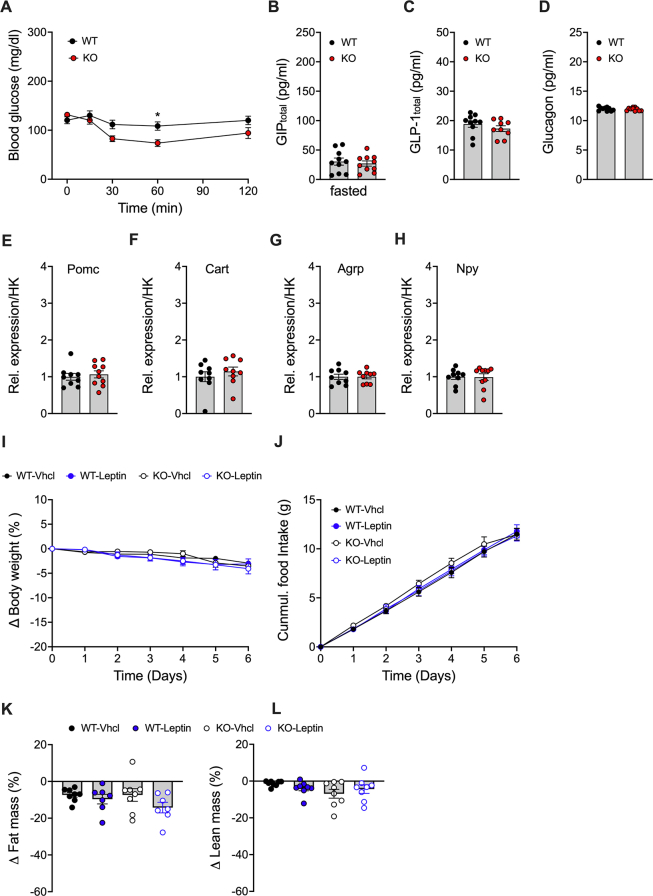
Plasma and qPCR analysis in male DIO *Lepr-Cre Gipr.***KO mice.** Intraperitoneal Insulin tolerance **(A)**, as well as fasted plasma levels of total GIP **(B)**, total GLP-1 **(C)**, and glucagon **(D)** in 47-wk old mice (n=8-10 each group). Hypothalamic expression of *Pomc***(E)**, *Cart***(F)**, *Agrp***(G)** and *Npy***(H)** in 47-wk old mice (n=9-10 each group). Body weight (I), cumulative food intake (J), as well as fat and lean tissue mass (K and L) of 31-wk old DIO *Lepr* Cre *Gipr* KO and WT mice treated daily with human recombinant leptin (1 mg/kg/day) (n=8 each group). Data represent means ± SEM. Asterisks indicate ∗ p<0.05, ∗∗ p<0.01 and ∗∗∗ p<0.001. Longitudinal data in panels **A, I,** and **J** were analyzed using 2-way ANOVA and Bonferroni post-hoc analysis for individual time points. Bar graphs in panels **B-H** and **K, L** were analyzed using 2-tailed, 2-sided ttest. Data points in panels **B-H, K, L** represent independent biological replicates.

The observed expression profile of *Gipr* in the pancreas is consistent with the scRNA-seq data, showing that ∽75% of the *Gipr* positive cells in the embryonic pancreas co-express *Lepr* (Figure 3C). When chronically fed with a HFD, *Lepr-Gipr* KO mice show no difference in body weight or body composition relative to wildtype (*Lepr-Cre*^*+*^ *Gipr*^*wt/wt*^) controls (Figure 4E–G). No difference was observed in food intake (Figure 4H), energy expenditure (Figure 4I), locomotor activity (Figure 4J), fatty acid (FA) oxidation (Figure 4K), and substrate utilization (Figure 4L). But despite normal glucose tolerance (Figure 4M), DIO *Lepr-Gipr* KO mice show improved insulin sensitivity (Figure 4N and Supplementary Figure 3A), with normal levels of blood glucose (Figure 4O), but decreased levels of plasma insulin (Figure 4P) and HbA1c (Figure 4Q). Consistent with the decreased insulin levels (Figure 4P), we see glucose-stimulated insulin secretion decreased in DIO *Lepr-Gipr* KO mice (Figure 4R), and as demonstrated in isolated pancreatic islets, this effect is mediated by a diminished insulinotropic response to GIP but not to GLP-1 (Figure 4S). No differences were observed in plasma levels of triglycerides or cholesterol (Figure 4T,U). Also fasting plasma levels of total GIP, total GLP-1 and glucagon, as well as hypothalamic expression of *Pomc*, *Cart*, *Agrp* and *Npy,* were not different between DIO *Lepr-Gipr* KO mice and wildtype controls (Supplementary Figure 3B-H). We further find no difference in body weight, food intake or changes in fat or lean tissue mass between DIO *Lepr-Gipr* KO mice and wildtype controls following 6 days daily treatment with leptin (1 mg/kg/day) (Supplementary Figure 3I-L). Consistent with the phenotype of global [23] and neuronal [8] *Gipr* deficient mice*, Lepr-Gipr* KO mice show no difference in body weight, body composition, food intake, glucose or insulin tolerance, or fasting level of blood glucose and insulin relative to wildtype controls when fed with a standard chow diet (Figure 5A–H). Also, HbA1c, plasma levels of GLP-1, glucagon, triglycerides, as well as free fatty acids (FFA) and hypothalamic expression of *Pomc*, *Cart*, *Agrp* and *Npy,* were not different between chow-fed *Lepr-Cre Gipr* KO mice and wildtype controls (Figure 5I-Q). In summary, these data suggest that GIPR signaling in cells/neurons that express the leptin receptor does not play a major role in regulating body weight and food intake, but that deletion of *Gipr* in *Lepr* cells improves insulin tolerance and glucose handling, as assessed by HbA1c, under conditions of diet-induced obesity.

### 2.6 Preserved weight loss but impaired glycemic effects of acyl-GIP and GIPR:GLP-1R co-agonism in DIO Lepr-Gipr KO mice

**Figure 6:**
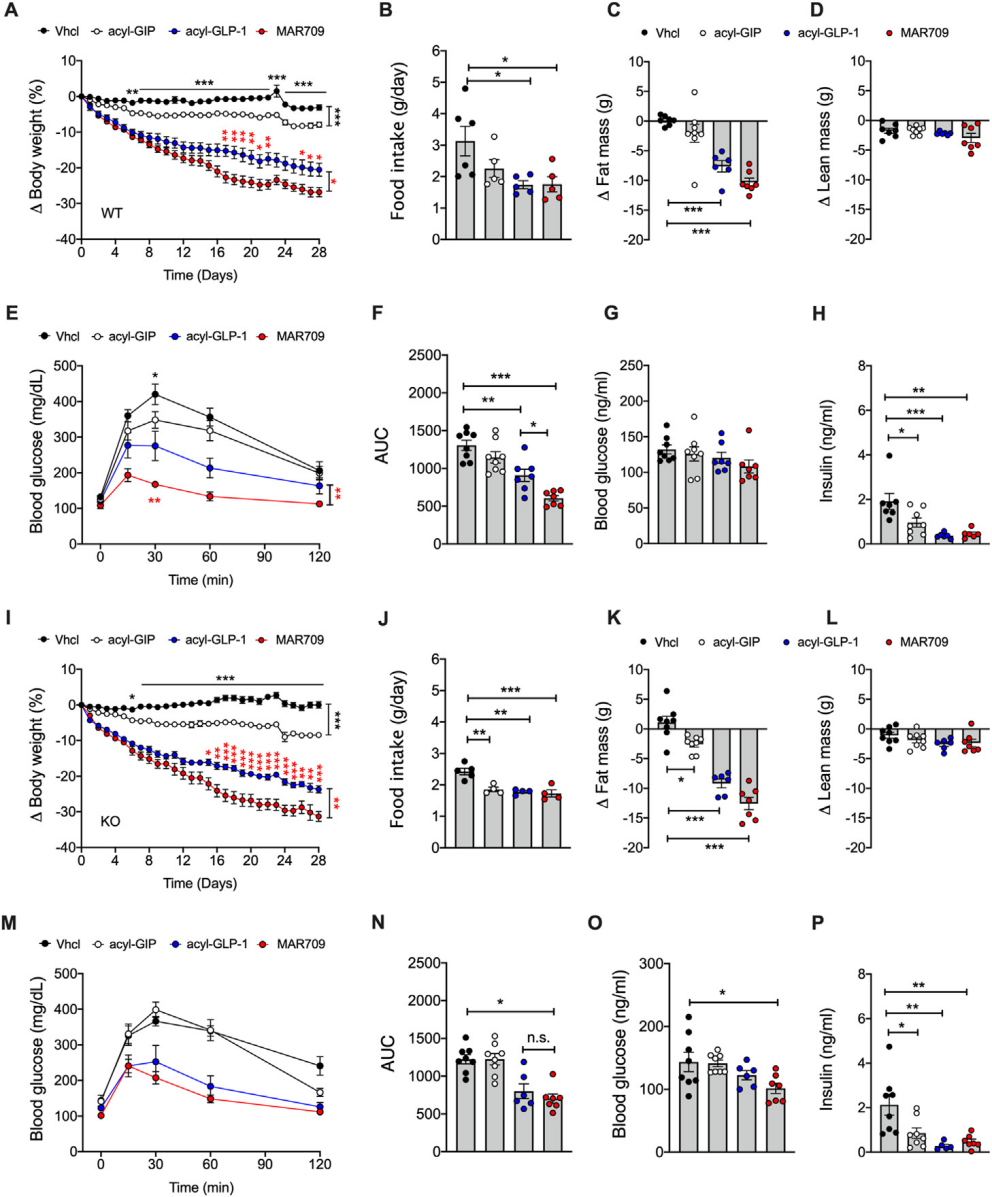
**Metabolic effects of GIP and GIPR:GLP-1R co-agonism in male DIO *Lepr*-*Gipr* KO mice.** Drug effects in wildtype **(A**–**H)** and *Lepr* Cre *Gipr* KO mice **(I–P)**. Body weight **(A)** and food intake **(B)** of DIO wildtype mice treated daily with acyl-GIP (100 nmol/kg) or 10 nmol/kg of either acyl-GLP-1 or MAR709 (n = 7–8 each group). Change in body composition (n = 6–8 each group) **(C, D)**, i.p. glucose tolerance **(E, F)**, as well as fasting levels of blood glucose **(G)** and insulin **(H)** after 23 days of treatment (**E-G**: n = 7–8 each group; **H**: 6-8 each group). Body weight **(I)** and food intake **(J)** of DIO *Lepr*-Cre *Gipr* KO mice treated daily with acyl-GIP (100 nmol/kg) or 10 nmol/kg of either acyl-GLP-1 or MAR709 (n = 6–8 each group). Change in body composition **(K, L),** i.p. glucose tolerance **(M, N)**, as well as fasting levels of blood glucose **(O)** and plasma insulin **(P)** after 23 days of treatment (**I–O:** n = 6–8 mice each group; **P:** n = 5–8 mice each group). Food intake **(B, I)** was assessed per cage in double-, or single-house mice (n = 6–8 mice each group). Data represent means ± SEM. Asterisks indicate ∗ p < 0.05, ∗∗p < 0.01 and ∗∗∗p < 0.001. Longitudinal data **(A, E, I, M)** were analyzed using 2-way ANOVA with time and treatment as co-variables and using Bonferroni post-hoc analysis for individual time-points. Bar graphs **(B-D, F–H, j-l, N–P)** were analyzed using 2-tailed, 2-sided ttest for comparison of acyl-GIP vs. Vhcl, and using 1-way ANOVA for comparison of Vhc, acyl-GLP-1 and MAR709. Data points in panels **B-D, F–H, J-L, N–P** represent independent biological replicates.

We next assessed whether the metabolic effects of GIP and GIPR:GLP-1R co-agonism depend on GIPR signaling in *Lepr* cells/neurons. In DIO wildtype mice, acyl-GIP (100 nmol/kg/day) decreased body weight with only slight inhibition of food intake (Figure 6A,B), and without overt changes in body composition (Figure 6C,D), glucose tolerance (Figure 6E,F) and blood glucose (Figure 6G), but decreased level of plasma insulin (Figure 6H). In DIO *Lepr-Gipr* KO mice, acyl-GIP decreased body weight with comparable efficacy relative to wildtype controls (Figure 6I), but with even greater inhibition of food intake (Figure 6J). Weight loss induced by acyl-GIP was paralleled by a decrease in fat mass (Figure 6K), without changes in lean body mass (Figure 6L), glucose tolerance (Figure 6M,N) or blood glucose (Figure 6O), but with decreased level of plasma insulin (Figure 6P). In both DIO wildtype and *Lepr-Gipr* KO mice, MAR709 (10 nmol/kg/day) led to greater decrease in body weight relative to mice treated with an equimolar dose of a pharmacokinetically (PK)-matched acyl-GLP-1 control (Figure 6A,I), and this was paralleled by a slightly greater decrease in food intake and fat mass (Figure 6B,C,J,K), without changes in lean body mass (Figure 6D,K). As expected from our previous studies [8], MAR709 improved glucose tolerance in wildtype mice with superior potency relative to the PK-matched acyl-GLP-1 control (Figure 6E,F). In the *Lepr-Gipr* KO mice, however, MAR709 lost its superior potency over acyl-GLP-to improve glucose tolerance (Figure 6M,N). MAR709 nonetheless decreased blood glucose in *Lepr-Gipr* KO mice (Figure 6O), with decreased level of plasma insulin that were comparable to acyl-GLP-1 (Figure 6H,P). Collectively, these data indicate that acyl-GIP and MAR709 decrease body weight and food intake independent of GIPR signaling in *Lepr* cells/neurons, but that the glycemic effects of GIPR:GLP-1R co-agonism crucially dependent on GIPR signaling in *Lepr* cells.

## 3 Discussion

In this manuscript, we assessed whether GIPR agonism affects energy and glucose metabolism via GIPR signaling in cells that express the leptin receptor. Using our own and publicly available RNAseq repositories [15,17], we show only limited co-expression of *Gipr* and *Lepr* in the hypothalamus, but considerable co-expression in the endocrine embryonic pancreas, including the beta cells. Consistent with the co-expression profile of *Gipr* and *Lepr*, we find that *Gipr* expression in *Lepr*-*Gipr* KO mice is largely preserved in the *Gipr* enriched hypothalamus, but substantially blunted in the pancreas. In line with this notion, we show that mice with *Lepr*-Cre-mediated deletion of *Gipr* have a normal body weight and food intake when fed with a HFD, but improved insulin sensitivity and decreased HbA1c. The improvement of glycemic control in DIO *Lepr-Gipr* KO mice is likely attributed to the demonstrated decrease of *Gipr* expression in the pancreas, which is consistent with the phenotype of mice with global [23] and beta cell specific [21] *Gipr* deletion. Of note, consistent with the phenotype in the DIO *Lepr-Gipr* KO mice, glucose tolerance of beta cell specific *Gipr* KO mice is indistinguishable from wildtype controls when fed with a HFD [21]. In contrast to previous studies showing that acyl-GIP and the dual incretin receptor agonist MAR709 lose their ability to decrease body weight and food intake in mice with deletion of *Gipr* in either the CNS [8], or specifically in GABAergic cells/neurons [9], we find acyl-GIP and MAR709 to retain their ability to decrease body weight and food intake in DIO *Lepr*-*Gipr* KO mice. Consistent with this, we see that MAR709 decreases body weight in DIO *Lepr*-*Gipr* KO mice with equal potency as in wildtype mice, and with superior efficacy over a PK-matched acyl-GLP-1 control. These data collectively indicate that GIP affects body weight and food intake independent of GIPR signaling in *Lepr* cells/neurons. Interestingly, acyl-GIP nonetheless stimulates cFos neuronal activation in the ARC, including a subpopulation of POMC neurons. These data are consistent with previous data, showing that acyl-GIP induces cFos neuronal activation in the hypothalamus [8], and that *Gipr* is expressed in a subpopulation of POMC neurons [11,15]. Whether GIP affects energy metabolism via this subpopulation of POMC neurons warrants clarification. Notably, while we show that GIPR signaling in *Lepr* cells does not affect body weight or food intake in mice chronically exposed to HFD, we see MAR709 in *Lepr-Gipr* KO mice to lose its superior efficacy to improve glucose tolerance relative to a PK-matched acyl-GLP-1 control. These data again support the notion that MAR709 is a GIPR:GLP-1R co-agonist that improves glucose tolerance by acting at both target receptors in rodents, and further that GIPR signaling in *Lepr* cells regulates glucose metabolism. Notably, and in contrast to previous studies showing that β-cell ablation of *Gipr* enhances the insulinotropic effect GLP-1 in isolated islets [21], we do not see acyl-GLP-1 to exhibit greater potency for glucose tolerance improvements in DIO *Lepr*-*Gipr* KO mice. The lack of greater GLP-1 responsiveness in DIO *Lepr*-*Gipr* KO mice might be attributed to the duration of drug treatment, and/or the incomplete deletion of *Gipr* in the β-cells (only ∽75% of the embryonic pancreatic cells express *Gipr* and *Lepr*). Collectively, we here show for the first time that GIPR signaling in cells that express the leptin receptor are not implicated in the regulation of body weight and food intake, but that lack of GIPR signaling in *Lepr* cells diminishes the glycemic beneficial effects of acyl-GIP and of GIPR:GLP-1R co-agonism under conditions of diet-induced obesity. Limitations of this study include that there are no validated GIPR selective antibodies available to assess co-staining of GIPR with target cells/neurons of interest, such as e.g. LEPR or GLP-1R. Another limitation is that current scRNAseq approaches do not allow to distinguish between different *Lepr* isoforms, and hence only refer to the full-lenghts *Lepr* transcript. Questions that remain to be addressed in future studies include whether the here shown acyl-GIP mediated activation of POMC neurons is implicated in the regulation of energy and glucose metabolism, and whether also hindbrain GIP responsive neurons affect systemic energy metabolism via projections to the hypothalmus.

## 4 Methods

### 4.1 Animals and housing conditions

Experiments were performed in accordance with the Animal Protection Law of the European Union after permission by the Government of Upper Bavaria, Germany. Mice were double- or single-housed and fed ad libitum with either chow (#1314, Altromin, Germany) or high-fat diet (D12331, Research Diets, New Brunswick, USA) under constant ambient conditions of 22 ± 2 °C with constant humidity (45–65%) and a 12 h/12 h light/dark cycle. Leptin receptor-Cre mice (Jackson Laboratory; #008320) [22] were back-crossed to C57BL/6J for >10 generations. C57BL/6J *Gipr*^*flx/flx*^ mice [20,21] were crossed with *Lepr-Cre+* mice (Jackson Laboratory; #008320) to obtain *Lepr-Cre+ Gipr*^*flx/flx*^ (*Lepr*-*Gipr* KO). *Lepr-Cre+ Gipr*^*wt/wt*^ were used as wildtype (WT) controls. For metabolic phenotyping, age-matched mice were double-housed and grouped based on their genotype. Body composition was analyzed using a magnetic resonance whole-body composition analyzer (EchoMRI, TX, USA). The number of biological replicates is stated in the figure legends. No animals were excluded from the analysis, unless animal welfare reasons demanded exclusion of singular animals (e.g. due to fighting injury or dermatitis). Most, but not all investigators were blinded for the treatment conditions.

### 4.2 Pharmacological studies

For the pharmacological studies, age-matched male mice were randomly distributed according to their genotype in groups of equal body weight and body composition. Mice were subsequently treated with a validated long-acting acyl-GIP (IUB0271, Supplementary Figure 2), which we previously showed to decrease body weight via neuronal GIPR signaling [8]. As a comparator for the validated GIPR:GLP-1R co-agonist MAR709 [2,8], we used the GLP-1R-selective, and pharmacokinetically (PK)-matched, acyl-GLP-1 backbone of MAR709 (IUB1746, Supplementary Figure 2). Human recombinant leptin was purchased from R&D Systems Inc., Abingdon, UK (#398-LP-05M). Mice were fed with a high-fat diet (HFD) for at least 20 weeks prior to start of the studies, followed by 28 days of daily treatment with either vehicle (Vhcl), acyl-GIP (100 nmol/kg/day), or 10 nmol/kg of either acyl-GLP-1 or the GIPR:GLP-1R co-agonist MAR709.

### 4.3 Immunohistochemistry

For cFos staining, HFD-fed Pomc-GFP mice (Jackson laboratories, #009593) [14] were anesthetized with CO_2_ 1.5 h after single s.c. administration of the acyl-GIP (30 nmol/kg), and transcardially perfused with phosphate-buffered saline (PBS) followed by 4% neutral buffered paraformaldehyde (PFA). Brains were harvested and equilibrated in 30% sucrose for three days, and sliced on a cryostat in the coronal plane at 30 μm. Slices were washed 5 times with 0.5% Triton X-100 in tris-buffered saline (TBS) followed by 1 h block with SUMI (0.25% gelatin and 0.5% TritonX-100 in TBS). After blocking, slices were incubated overnight with primary antibody anti-cFOS (rabbit polyclonal 226003, 1:2000, Synaptic System, Goettingen, Germany) in SUMI at 4 °C. After 5 times wash in TBS, slices were incubated 1 h with Alexa Fluor 568 donkey-anti-rabbit (1:1000, Molecular Probes, Life Technologies GmbH, Darmstadt, Germany) secondary antibody. After several washes, slices were mounted on gelatin-pre-coated glass slides, and cover-slipped for image quantification. ImageJ was applied for quantifying cFos postivie cells and cFos-POMC co-localized cells. Images of single focal planes were captured at 20× magnification by a Leica SP5 scanning confocal microscope. The number of cFos positive nuclei within the hypothalamic area was determined according to the Allen mouse brain atlas and the analyses were performed without previous knowledge of the experimental group.

### 4.4 Fasting glucose, ipGTT, ipITT

Plasma levels of glucose and insulin were measured after 6 h fasting. For assessment of glucose tolerance, glucose was administered i.p at a dose of 1.5 g/kg. For assessment of insulin tolerance, insulin (Humalog; Eli Lilly and Co, USA) was injected i.p at a dose of 0.75 U/kg.

### 4.5 Pancreatic islets isolation

Mice were sacrificed by cervical dislocation and clamping the bile duct and perfuse the collagenase P (Roche) solution immediately. In brief, 1 ml of cold collagenase P solution (1 mg/ml dissolved in G-solution (HBSS (Lonza) + 1% BSA (Sigma–Aldrich)) was injected into the bile duct and the perfused pancreas was consequently dissected. Tissue pieces were incubated in a 15 ml Falcon tube with 1 ml of collagenase P solution, which is same as the injection solution, for 15 min at 37 °C with a strong shaking in the middle of incubation. Then, 12 ml of the cold G-solution was filled into the falcon tubes with samples, followed by centrifugation at 1,620 rpm at room temperature. Pellet was washed with 10 ml of the G-solution. After washing step with the G-solution, the pellets were re-suspended in 5.5 ml of gradient solution – 15% of Optiprep (5 ml 10% RPMI (Lonza) + 3 ml of 40% Optiprep which diluted from 60 % Optiprep with G-solution (Sigma–Aldrich) per sample), and placed on top of 2.5 ml of the gradient solution. To form a 3-layer gradient, 6 ml of the G-solution was added on the top. Samples were then incubated for 10 min at room temperature before centrifugation at 1,700 rpm. Finally, the interphase between the upper and the middle layers of the gradient was harvested and was filtered through a 70 μm nylon filter then washed with the G-solution. Islets were handpicked by a micropipette under the microscope and cultured in RPMI 1640 medium overnight.

### 4.6 *Ex vivo*Glucose stimulated insulin secretion (GSIS) from pancreatic islets

Prior to GSIS, culture medium was removed and islet microtissues were washed twice with Krebs Ringer Hepes Buffer (KRHB; 131 mM NaCl, 4.8 mM KCl, 1.3 mM CaCl_2_, 25 mM HEPES, 1.2 mM KH_2_PO_4_, 1.2 mM MgSO_4_, 1% BSA) containing 2.8 mM, glucose and equilibrated for 1 h in the same solution. The supernatant was collected as a sample under low glucose condition, and islets were incubated for another 1 h at 37 °C with KRHB containing 16.7 mM glucose and supplements as above. The supernatant was collected as a sample under high glucose condition and stored at −20 °C. For drug-induced insulin secretion, acyl-GIP or acylGLP-1 were diluted in 1× KRK buffer with 20 mM glucose to reach a concentration of 50 nM. Cells were subsequently treated with either acyl-GIP or acyl-GLP-1 for 45 min. The remaining islets were lysed in 500 ul of Acid-Ethanol (70% Ethanol with 1.5% HCl 12N) using the sonicator and incubated at 4 °C overnight. Lysed cells were centrifuged (7,000 rpm, 4 °C, 10 min), and the supernatant was transferred into a new tube and stored at −20 °C. Insulin concentrations were determined using the Mouse insulin ELISA (AppliChem), and secreted insulin was normalized to total insulin content.

### 4.7 *Oral* glucose-stimulated insulin secretion (GSIS)

Glucose was given orally at a dose of 4 g/kg body weight in 6 h fasted mice, followed by blood sampling at time points 0, 2, 5, 15 and 30 min after glucose administration.

### 4.8 Plasma analysis

Blood samples were collected in EDTA tubes and centrifuged for 10 min at 3000×*g* and 4 °C. Plasma total immunoreactive insulin was measured using commercially available ELISA kits from Crystal Chem, Zaandam, Netherlands (# 90080). Total GIP was measured using ELISA (Sigma Aldrich; #EZRMGIP-55K). Plasma triglycerides was determined using commercially available kits from Wako Chemical (# 290-63701). Total cholesterol was determined using kits from Thermofisher scientific, MA, USA (#10178058), Plasma NEFA levels were determined by enzymatic assay using reagents from Wako Chemicals, Japan (#917979 and #91898). All ELISAs were performed according to the manufacturer's instruction.

### 4.9 Indirect calorimetry

Energy expenditure, respiratory exchange ratio (RER), and locomotor activity were assessed in single-house mice using a climate-controlled indirect calorimetric system for 137 h preceded by 24 h of acclimatization (TSE Phenomaster, TSE Systems GmbH, Germany). Data for energy expenditure were analyzed using ANCOVA with body weight as a covariate as previously suggested [24,25]. Fatty acid oxidation (kcal/h) was calculated by the formula: energy expenditure (kcal/h) x (1-RER)/0.3.

### 4.10 RNA extraction and gene expression analysis

Total RNA was prepared using RNeasy Kit (QIAGEN, Hilden, Germany) according to manufacturer's instructions. cDNA synthesis was performed using QuantiTect Reverse Transcription Kit (QIAGEN, Hilden, Germany) according to manufacturer's instructions. Gene expression was profiled using RT-qPCR-based (qPCR) techniques using SYBR green (Thermo Fisher Scientific, Erlangen, Germany). The relative expression of the selected genes was measured using a Quantstudio 7 flex cycler (Applied biosystems, CA, USA). The relative expression levels of each gene were normalized to the housekeeping gene Hypoxanthine Phosphoribosyl transferase (*Hprt*). Primer sequences were *Hprt*-F: 5′-CCC TGG TTA AGC AGT ACA GCC CC-3′, *Hprt*-F: 5′-AGT CTG GCC TGT ATC CAA CAC TCG-3′, *Agrp*-F: 5′-CGG CCA GAA CCT CTG TAG-3′, *Agrp*-R: 5′-CTC ATC CCC TGC CTT TGC-3‘, *Pomc*- F: 5′-CAT TAG GCT TGG AGC AGG TC-3′, *Pomc*-R: 5′-TCT TGA TGA TGG CGT TCT TG-3′, *Cart*- F: 5′- CGA GAA GAA GTA CGG CCA AG-3′, *Cart*-R: 5′-GGA ATA TGG GAA CCG AAG GT-3′, *Gipr*-F: 5′-GTG TCC ACG AGG TGG TGT TT-3′, *Gipr*-R: 5′- CCG ACT GCA CCT CTT TGT TG-3′; *Npy*-F: 5′-TGG ACT GAC CCT CGC TCT AT-3′, *Npy*-R: 5′- TGT CTC AGG GCT GGA TCT CT -3′.

### 4.11 Fluorescence-activated cell sorting (FACS) and single-nucleus RNA sequencing (snRNA-seq) of hypothalamic LepR-positive nuclei

To isolate Lepr-positive hypothalamic nuclei for single nucleus RNA sequencing, Sun1-sfGFP-Myc+ mice (B6;129-Gt(ROSA)26Sortm5(CAG-Sun1/sfGFP)Nat/J; JAX #021039) were crossed with Lepr-Cre^+^ mice (B6.129-Leprtm3(cre)Mgmj/J; JAX #032457). Heterozygous Lepr-Cre:R26-CAG-LSLSun1-sfGFP-Myc mice were group-housed on a 12:12-h light–dark cycle at 23 °C with ad libitum food intake until an average age of 20 weeks. Hypothalami were then excised and subjected to nuclear isolation using a dounce homogeniser as previously described [26]. Nuclear pellets were resuspended in buffer (PBS, 0.15 mM spermine, 0.5 mM spermidine, 0.4 units Protector RNase inhibitor, 0.4% IGEPAL-630, 1% BSA) supplemented with 1 μg/μL DAPI. Two samples per condition were pooled, assessed for nuclear integrity using a Zeiss microscope (Axio Scope, Zeiss, Germany), and subjected to FACS (FACS-Aria III, BD Biosciences). Sorting was performed using a 70 μm nozzle. Doublet discrimination and DAPI staining were used for appropriate gating of single nuclei, signals on the green (FITC) channel of Lepr negative samples served as negative control. Lepr positive nuclei were sorted into 5 μl of nuclei buffer reaching a final concentration of 0.5% BSA and 0.2 U/μl Protector RNase inhibitor. Next, the Chromium Next GEM Automated Single Cell 3′ Reagent Kit v3.1 (10× Genomics, #1000268) was utilized to prepare libraries in accordance with the manufacturer's instructions. The resulting libraries underwent 150-bp paired-end sequencing of read 2 on a HiSeq4000 (Illumina), snRNA-seq data analyses were performed using the Seurat 4.1 package [27].

### 4.12 Single cell RNA sequencing in embryonic pancreas

Cells expressing*Lepr* and *Gipr* in the embryonic pancreas and hypothalamus were identified in various data sets available in interactive data viewers for single-cell data: UCSC Cell Browser [28] and CELLxGENE. To identify cell types that express *Gipr* and/or *Lepr* in mouse embryonic pancreas [17] (Figure 3C), metadata for cells expressing each gene were downloaded and cells belonging to different clusters were identified by cell ID. The percentage of cells expressing *Lepr* and/or *Gipr* within different neuronal populations was calculated with CELL x GENE based on the HypoMap repository [15] by subsetting to cells expressing *Gipr, Lepr*, *Agrp*, *Pomc, Slc32a1* or Slc17a6.

### 4.13 Single nucleus RNA sequencing in hindbrain

The DVC snRNAseq dataset [16] was analyzed using scanpy [29]. The authors original pre-processing and cell-type annotations were used. Cells with at least 1 unique molecular identifier (UMI) count of *Gipr*, *Vgat*, or *Lepr* were considered to express that gene.

### 4.14 Replicates, randomization and blinding

*In vivo* studies were performed in male age-matched mice that were randomly distributed into groups of equal body weight and body composition. The number of independent biological samples per group is stated in the figure legends. No animals were excluded from the studies unless health issues demanded exclusion of single mice (e.g., due to fighting injuries). For *in vivo* studies, drugs were aliquoted by a lead scientist in number-coded vials and most, but not all, handling investigators were blinded to the treatment condition. Analyses of glucose and insulin tolerance were performed by experienced research assistants which did not know prior treatment conditions.

### 4.15 Statistical analysis

For animal studies, sample sizes were calculated based on a power analysis assuming that a greater or equal (≥) 5 g difference in body weight between genotypes can be assessed with a power of ≥75% when using a 2-sided statistical test under the assumption of a standard deviation of 3.5 and an alpha level of 0.05. Statistical analyses were performed using the statistical tools implemented in GraphPad Prism8 (version 8.3.0). Differences between groups were assessed by Student's 2-sided 2-tailed t-test, 1-way ANOVA or 2-way ANOVA with time and genotype as co-variants followed by Bonferroni's *post-hoc* multiple comparison testing for individual time points. All results are given as mean ± SEM. *P* < 0.05 was considered statistically significant, with ∗ indicating p < 0.05, ∗∗ indicating p < 0.01 and ∗∗∗ indicating p < 0.001. Differences in energy expenditure were calculated using ANCOVA with body weight as co-variate using SPSS (version 24) as previously suggested [24,25]. No data were excluded from the analysis unless identification of singular outlier using Grubbs test.

### 4.16 Study approval

Experiments were performed in accordance with the Animal Protection Law of the European Union after permission by the Government of Upper Bavaria, Germany.

## CRediT authorship contribution statement

**Seun Akindehin:** Writing – original draft, Investigation, Formal analysis, Data curation. **Arkadiusz Liskiewicz:** Supervision, Investigation, Formal analysis, Data curation. **Daniela Liskiewicz:** Investigation, Formal analysis, Data curation. **Miriam Bernecker:** Investigation, Formal analysis, Data curation. **Cristina Garcia-Caceres:** Investigation, Formal analysis, Data curation. **Daniel J. Drucker:** Writing – review & editing, Methodology. **Brian Finan:** Writing – review & editing, Project administration, Methodology, Formal analysis. **Gerald Grandl:** Writing – review & editing, Supervision, Formal analysis. **Robert Gutgesell:** Writing – review & editing, Software, Formal analysis. **Susanna M. Hofmann:** Writing – review & editing, Investigation, Formal analysis, Data curation. **Ahmed Khalil:** Formal analysis, Data curation. **Xue Liu:** Formal analysis, Investigation. **Perla Cota:** Data curation, Formal analysis. **Mostafa Bakhti:** Conceptualization, Data curation. **Oliver Czarnecki:** Conceptualization, Data curation. **Aimée Bastidas-Ponce:** Conceptualization, Data curation. **Heiko Lickert:** Writing – review & editing, Supervision, Formal analysis. **Lingru Kang:** Conceptualization, Data curation. **Gandhari Maity:** Supervision, Investigation, Formal analysis. **Aaron Novikoff:** Supervision, Formal analysis, Data curation. **Sebastian Parlee:** Supervision, Investigation, Formal analysis. **Ekta Pathak:** Formal analysis, Data curation. **Sonja C. Schriever:** Supervision, Investigation, Formal analysis. **Michael Sterr:** Formal analysis, Data curation. **Siegfried Ussar:** Conceptualization, Data curation. **Qian Zhang:** Methodology, Investigation, Formal analysis, Data curation. **Richard DiMarchi:** Writing – review & editing, Supervision, Resources, Methodology. **Matthias H. Tschöp:** Writing – review & editing, Supervision. **Paul T. Pfluger:** Supervision, Investigation, Formal analysis, Data curation. **Jonathan D. Douros:** Writing – review & editing, Supervision, Resources, Methodology, Investigation, Formal analysis, Data curation. **Timo D. Müller:** Writing – review & editing, Writing – original draft, Validation, Supervision, Resources, Investigation, Funding acquisition, Formal analysis, Conceptualization.

## Declaration of competing interest

MHT is a member of the scientific advisory board of ERX Pharmaceuticals, Cambridge, Mass. He was a member of the Research Cluster Advisory Panel (ReCAP) of the Novo Nordisk Foundation between 2017 and 2019. He attended a scientific advisory board meeting of the Novo Nordisk Foundation Center for Basic Metabolic Research, University of Copenhagen, in 2016. He received funding for his research projects by 10.13039/501100004191Novo Nordisk (2016–2020) and Sanofi-Aventis (2012–2019). He was a consultant for Bionorica SE (2013–2017), Menarini Ricerche S.p.A. (2016), and Bayer Pharma AG Berlin (2016). As former Director of the Helmholtz Diabetes Center and the Institute for Diabetes and Obesity at Helmholtz Zentrum München (2011–2018), and since 2018, as CEO of Helmholtz Zentrum München, he has been responsible for collaborations with a multitude of companies and institutions, worldwide. In this capacity, he discussed potential projects with and has signed/signs contracts for his institute(s) and for the staff for research funding and/or collaborations with industry and academia, worldwide, including but not limited to pharmaceutical corporations like Boehringer Ingelheim, Eli Lilly, 10.13039/501100004191Novo Nordisk, Medigene, Arbormed, BioSyngen, and others. In this role, he was/is further responsible for commercial technology transfer activities of his institute(s), including diabetes related patent portfolios of Helmholtz Zentrum München as, e.g., WO/2016/188932 A2 or WO/2017/194499 A1. 10.13039/100004971MHT confirms that to the best of his knowledge none of the above funding sources were involved in the preparation of this paper. TDM and K.S. receive research funding by 10.13039/501100004191Novo Nordisk but these funds are unrelated the here described work. DJD has received speaking and consulting fees from 10.13039/100004334Merck and 10.13039/501100004191Novo Nordisk Inc and consulting fees from Forkhead Biopharmaceuticals and Kallyope Inc. R.D.D is a co-inventor on intellectual property owned by 10.13039/100006733Indiana University and licensed to 10.13039/501100004191Novo Nordisk. He was previously employed by Novo Nordisk. P.J.K, S.M., and B.F. are current employees of Novo Nordisk. TDM receives funding from 10.13039/501100004191Novo Nordisk and received speaking fees within the last 3 years from 10.13039/501100004191Novo Nordisk, Eli Lilly, 10.13039/100004325AstraZeneca, 10.13039/100004334Merck, Berlin Chemie AG, and Mercodia.

## Data Availability

Data will be made available on request.
